# The severity and frequency distribution of endometriosis subtypes at different ages: a model to understand the natural history of endometriosis based on single centre/single surgeon data

**DOI:** 10.52054/FVVO.13.3.028

**Published:** 2021-09-24

**Authors:** P.R. Koninckx, A Ussia, A Wattiez, L Adamyan, D.C. Martin, S Gordts

**Affiliations:** Latifa Hospital, Dubai, United Arab Emirates; ObGyn KULeuven Belgium; Gruppo Italo Belga, Villa Del Rosario Rome, Italy; Università Cattolica, Rome, Italy; Department of Obstetrics and Gynaecology, University of Strassbourg, France; Department of Operative Gynecology, Federal State Budget Institution V. I. Kulakov Research Centre for Obstetrics, Gynecology, and Perinatology, Ministry of Health of the Russian Federation, Moscow, Russia; and Department of Reproductive Medicine and Surgery, Moscow State University of Medicine and Dentistry, Moscow, Russia; University of Tennessee Health Science Centre, Memphis Tennessee, USA, Institutional Review Board, Virginia Commonwealth University, Richmond, Virginia, USA; Leuven Institute for Fertility & Embryology, Leuven Belgium; University of Oxford-Honorary consultant, UK; Moscow State Univ

**Keywords:** Endometriosis, endometriosis natural history, endometriosis growth, endometriosis prevention

## Abstract

**Background:**

The natural history of endometriosis is poorly understood. Animal models are inadequate, repeat laparoscopies are not acceptable and epidemiological studies on endometriosis are complicated.

**Objective:**

To study the natural history of endometriosis by reviewing the frequency distribution of clinical subtypes of endometriosis lesions at different ages in women undergoing surgery.

**Materials and methods:**

Surgical findings of all women (n=2086) undergoing laparoscopy for pelvic pain and endometriosis between 1988 and 2011 at University Hospital Gasthuisberg were analysed.

**Main outcome measures:**

The severity of subtle, typical, cystic and deep endometriosis in adult women, with or without a pregnancy, as estimated by their pelvic area and their volume.

**Results:**

The number of women undergoing a laparoscopy increased up to 28 years of age and decreased thereafter. Between 24 and 44 years, the severity and relative frequencies of subtle, typical, cystic and deep lesions did not vary significantly. The number of women younger than 20 years was too small to ascertain the impression of less severe lesions. The severity of endometriosis lesions was not less in women with 1 or more previous pregnancies or with previous surgery. There was no bias over time since the type and severity of endometriosis lesions remained constant between 1988 and 2011.

**Conclusions:**

Severity of endometriosis does not increase between 24 and 44 years of age, suggesting that growth is limited by intrinsic or extrinsic factors. Severity was not lower in women with a previous pregnancy.

**What is new:**

Considering the time needed for lesions to become symptomatic together with the diagnostic delay, the decreasing number of laparoscopies after age 28 is compatible with a progressively declining risk of initiating endometriosis lesions after menarche, the remaining women being progessively less susceptible.

## Introduction

The pathophysiology and the natural history of endometriosis are poorly understood. Endometriosis was defined histologically in 1925 as endometrium like cells outside the uterus (Sampson, 1925). One hypothesis was that the disease started with the implantation of endometrial cells from retrograde menstruation or after transport by the bloodstream. Previously, in the 19 th century similar lesions, then called adenomyoma (Rokitansky, 1860; Cullen, 1897), were thought to originate from metaplasia of mesothelial cells. This concept of metaplasia has remained since not all lesions could be explained by implantation. Later metaplasia of Müllerian remnants was proposed (Batt and Smith, 1989). These concepts, based on the contemporary knowledge of histology and endocrinology, have been challenged by the fast increasing understanding of molecular biological pathways, epigenetics and genetics. Endometriosis lesions were shown to be different for many aspects as clonality (Anglesio et al., 2015; Suda et al., 2018), endocrinology (Bulun et al., 2015; Flores et al. 2018) or cancer driver mutations (Yoo et al., 2017; Guo, 2018a; Bulun et al., 2019). To integrate these observations the genetic-epigenetic (GE) theory was proposed, postulating that a cumulative set of GE incidents need to reach a threshold (Koninckx et al., 2019a), to initiate progression to typical, cystic or deep endometriosis.

The subsequent growth and development of endometriosis lesions vary with the local endocrine, immunologic and cellular environment and because of the repetitive trauma of bleeding in the lesions (Canis et al., 2017; Guo, 2018b) and fibrosis (Vigano et al., 2020). Since the growth of clonal lesions varies with their specific set of GE incidents, we suggested that the initiation and the subsequent growth need to be separated to understand endometriosis (Koninckx et al., 2020).

The natural history of endometriosis lesions remains poorly documented. There is no adequate animal model and in the human repetitive laparoscopies are not acceptable. The epidemiology is complicated (Koninckx et al., 2021a) by the different views of subtle lesions. These can be viewed as early and active lesions following implantation or metaplasia, which mostly disappear spontaneously (Wiegerinck et al., 1993). However, some of them progress to severe lesions. That the latter are clonal suggests that a GE incident occurred, similar to cancers, because of oxidative (McGranahan and Swanton, 2017) or infectious stress (Koninckx et al., 2019b). It is unclear how the growth of lesions is regulated, and whether lesions keep growing (Redwine et al. 1991; Fedele et al. 2004) or stop growing after some time. Indirect clinical and histopathological data suggest that the growth of most typical, cystic and deep lesions is limited. They can be histologically inactive, macroscopically fibrotic and clinically no longer actively growing when diagnosed (Koninckx et al., 2019a). Without repetitive laparoscopies, it is not clear whether medical treatment or pregnancies prevent the progression of endometriosis lesions (Koninckx et al., 2018; Knox et al., 2019).

Understanding the pathophysiology and the natural history of endometriosis is important for its management (Gordts et al., 2017). Since the non-invasive diagnosis of initial endometriosis lesions is limited, management balances between early laparoscopy and surgery versus postponing a laparoscopy and surgery by medical treatment as long as clinically reasonable. The former has the risk of recurrences and repeat surgery (Redwine, 1991; Shakiba et al., 2008; Yeung et al., 2011; Martin, 2015); the latter carries the risk that lesions can grow with more damage and more difficult surgery later. This dilemma is especially difficult for endometriosis in adolescence since a young age does not facilitate clinical diagnosis.

To understand the natural history of endometriosis, we reviewed the frequency distribution of clinical subtypes of endometriosis lesions at different ages in women undergoing surgery between 1988 and 2011 at University Hospital Gasthuisberg, KULeuven, Belgium.

## Materials and methods

### The registration of endometriosis data

All surgeries of women operated by or under the supervision of PK between 1988 and 2011 in Leuven University Hospital Gasthuisberg, Belgium, were registered in a database. As described (Koninckx et al., 1991) descriptive data were entered immediately after surgery. For each endometriosis lesion, the type of the lesion, the localisation, the macroscopic appearance, the diameter, the depth and the number of similar lesions were registered. The depth and diameter were estimated by comparison with the openings at the end of the suction irrigation probe. The location was described as the ovarian fossa, the sidewall, the uterosacral ligaments, the vesico- uterine fold, the pouch of Douglas, the diaphragm, the sigmoid, the rectum, the ischial spine etc. The macroscopic appearances of endometriotic lesions were registered as white or red vesicles, polypoid or flame-like lesions, black puckered lesions in a white sclerotic area, as normal or as a peritoneal pocket. Peritoneal pockets were (erroneously) considered in Leuven as a type of endometriosisKoninckx et al., 2021b. The appearance was registered by one letter, permitting to enter any combination of lesions. The diameter and depth of deep lesions were estimated after excision. Lesions were considered deep endometriosis when the depth was more than 5 mm (Koninckx et al., 1993; Koninckx and Martin, 1994). To facilitate the calculation of the peritoneal surface and the volume of deep and cystic lesions, the diameter and the depth of deep lesions were approximated as if their peritoneal surfaces and their volumes were circular or spherical respectively. A superficial lesion of 5 by 15 mm thus was approximated and entered as a lesion of 10 mm in diameter, which is an approximation with a less than 5% error. For deep endometriosis lesions, depth and diameter estimations were made after ‘one bloc’ excision, after which most lesions had become more spherical by contraction. To reduce entry mistakes, the design of the database limited the entrance of numerical fields to a predefined range and character fields were limited to predefined initials for that field, such as P for pain and I for infertility and PI for pain and infertility or G for gunshot and W for a white area.

Over time existing items in the database were kept constant to avoid inconsistencies. However, new items were added. Around 1996, 2 fields were added specifying whether women had undergone previous surgery elsewhere. Whether it was the first or a repeat intervention in Leuven, was obvious from the database entries. It was not indicated whether women had been referred for surgery. Care was taken not to duplicate data. As an example, the age of the woman was calculated from days between the date of birth and the date of surgery.

### Frequency and severity of types of endometriosis at different ages

Women included in this study were all (n=2086) operated on for pain (P in the field ‘indication’) and having endometriosis (E in the field ‘endometriosis’). The first item registered in the database was the main indication for surgery being I(nfertility), P(ain), ovarian C(yst), F(ibroma), pelvic D(escent), cancer, bleeding disorders, uterine polyp, extra- uterine pregnancy (EUP) or others or a combination of these. The next item was the type of surgery (laparoscopy or laparotomy), and the third item was the diagnosis, being N(ormal), E(ndometriosis), A(dhesions), ovarian C(yst), F(ibroma), V(aricose veins), enterocoele, H(ydrosalpinx), extra-uterine pregnancy, I(nfection) or O(ther) or a combination of these. First the women with pain as the indication for surgery were selected (n=2806). Thus women with little or no pain such as a rare cystic ovarian endometriosis without pain, are not included since the indication for surgery was registered as an ovarian cyst. Important also is that pelvic pain as an indication for surgery was a clinical decision based on the severity, localization and radiation of pain and the clinical exam. In the absence of pain, the diagnosis of deep endometriosis by imaging was not an indication for surgery. However, this does not exclude that imaging by a transvaginal ultrasound examination or magnetic resonance imaging (MRI) could have confirmed the diagnosis of cystic ovarian or deep endometriosis. Medical therapy to postpone surgery was not given. Women with other lesions found by imaging but without pain were not included in this analysis. In these 2806 women with pain as an indication for surgery, 2086 women had endometriosis (74%), 117 a hydrosalpinx, 115 a myoma, 1633 adhesions and 113 women had a normal pelvis without an explanation of the pelvic pain. All 2086 women with endometriosis were included in this study, although 60 women also had a hydrosalpinx, 66 a myoma and 1254 adhesions.

This study evaluated in each woman the presence of subtle, typical, cystic ovarian and deep endometriosis lesions and their severity as estimated by their pelvic area and their volume. Considering the 14 possible combinations of subtle, typical, cystic and deep lesions (S, T, S, D, ST, SC, SD, TC, TD, CD, STC, STD, TCD and STCD) in one woman (Koninckx et al., 2021b), women were grouped according to the most severe lesion. Women were classified as subtle lesions (class S) if no other endometriosis lesions were present and as ‘typical lesions’ (class T) when typical but no cystic or deep endometriosis were present. Deep endometriosis (class D) was defined as a depth of more than 5 mm under the peritoneum (Koninckx et al., 1993; Koninckx and Martin, 1994). Women having cystic ovarian endometriosis (class C) and those having both cystic ovarian and deep endometriosis (class CD) were grouped separately. Some women with class T thus also could have subtle lesions and women with classes C and D typical and subtle lesions.

The study was registered with the Institutional review board of University Hospital Gasthuisberg (S63491) and the database had been kept as a General Data Protection Regulation compliant SAS file.

### Statistics

The data of the database (Visual FoxPro) were imported into SAS (SAS 1985). First, white and red vesicles and polypoid and flame-like lesions were grouped as subtle lesions. Gun-shot lesions with or without a white sclerotic area and with or without subtle lesions were grouped as typical lesions. The severity of each type of lesion was estimated by the peritoneal areas and their volumes, assuming a sphere for cystic and a cylinder for deep, typical and subtle lesions. Volumes of subtle lesions thus are probably slightly overestimated since their depth was arbitrarily considered as 1 mm.

A first analysis evaluated the relative frequencies of women classified as subtle or typical or cystic ovarian or deep or cystic and deep endometriosis. A second analysis evaluated the different types of lesions. To highlight the heterogeneity of endometriosis lesions and in order not to overlook eventual subgroups, Scatchard plots of the area, depth and volume of each type of endometriosis were plotted against the age of the women. To these graphs, cubic regression analyses with 95% confidence limits were added using proc gplot of SAS (SAS 1985). To visualise the effect of a pregnancy or the effect of being the first or second surgery, different colours were used. Significances between these groups were calculated by unpaired t-tests and visualised in box and whiskers plots. Women in whom large cystic endometriosis was marsupialised without any major surgery, were considered as a separate group and the surgery a few months later as the first surgery. To exclude that results might be the consequence of a systematic bias over time, the same data were also evaluated for their year of surgery (between 1988 and 2011). Statistical significance was evaluated also by Chi-square or regression analysis (Pearson and Spearman). P&0.05 was considered statistically significant.

## Results

In 1929 women, in whom data were available, 3.8% underwent a marsupialisation, 83.6% a first intervention and 12.6% a second intervention in Leuven. A first and second intervention were performed for women in classes S, T, C, D and C+D in 4.5%, 15.7%, 13.1%, 42.3%, and 16.2% and in 3.7%, 18.5%, 30.9%, 33.3, and 12.5% respectively. The women with the first intervention in Leuven had had a previous surgery elsewhere in 10.1%. Although the type, quality and completeness of this previous intervention could not be judged, surgery with excision of deep endometriosis had been extremely rare.

The incidence of endometriosis, in women undergoing laparoscopy for pain, decreased slightly (P<0.0001) between 20 and 44 years of age being for 2-year age groups between 20 and 44 years, 78, 82, 85, 83, 85, 79, 76, 78, 73, 72, 66, 56 and 52 per cent respectively.

**Figure 1:**
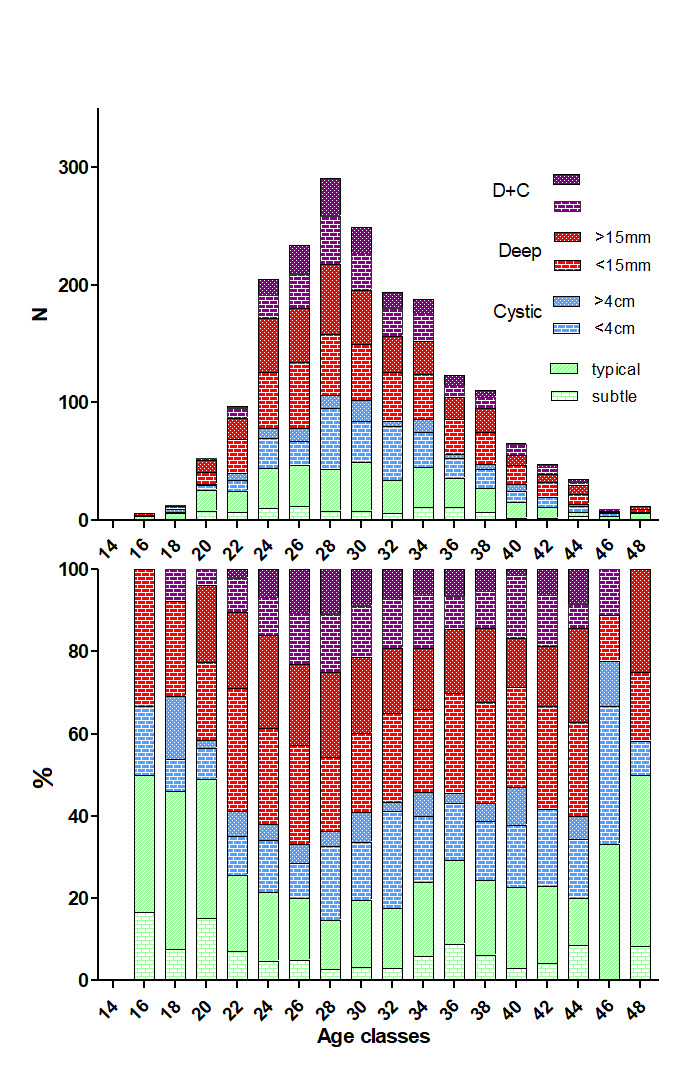
Number (upper) of women operated for superficial, cystic, deep, or cystic and deep endometriosis for ages between 14 and 50 years (total = 2086). Classes of 2 years are indicated, 14-15, 16-17 etc. In the lower graph, the relative frequencies of superficial, cystic ovarian (smaller and larger than 4cm), deep (less and deeper than 15mm), and deep (< and > 15mm) plus cystic ovarian endometriosis are shown.

The number of women undergoing laparoscopy for pain increased after puberty up to 28 years and declined thereafter (Figure 1). Only 13 women or 0.06%, underwent a laparoscopy before age 20, which is too small a group for meaningful conclusions. Only 58 women (2.8%) underwent a laparoscopy between 20 and 22 years of age. Considering the relative frequencies of lesions in the group of women between 14 and 28 years of age (Figure 1), the deep and cystic endometriosis became more frequent and superficial lesions were less frequent (Mantel-Haenszel P<0.0001). Between ages 24 and 44, the relative frequencies of each type of endometriosis, i.e. S (Subtle), T (Typical), C (Cystic ovarian), D (Deep) and CD (Cystic ovarian and Deep) were remarkably similar (NS). Also, the more severe deep (deeper than 15mm) and cystic (larger than 4cm) endometriosis did not become more frequent with age.

**Figure 2:**
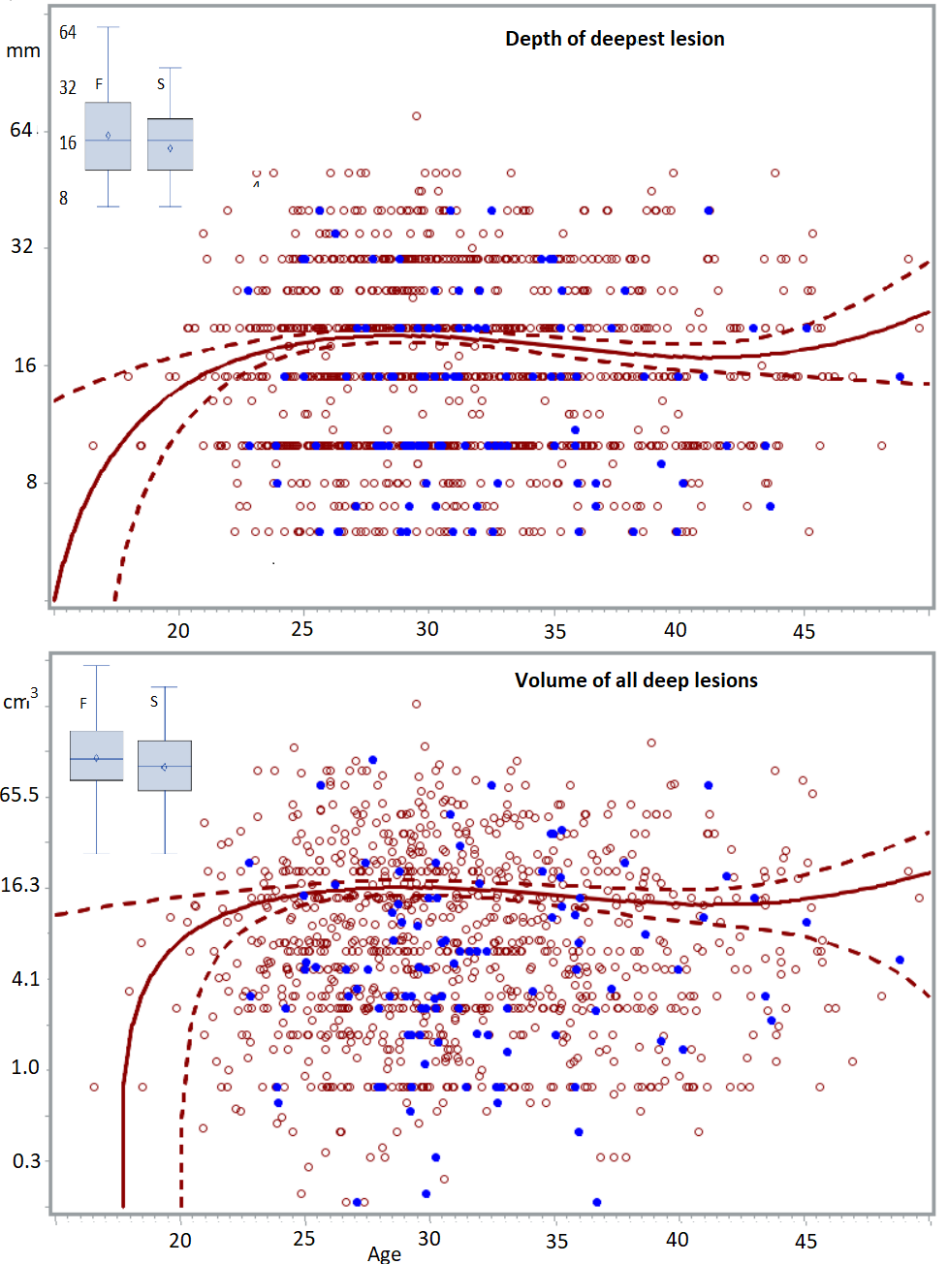
Depth of the deepest lesion (mm) and volume of all deep lesions (cm^3^) in women between 14 and 50 years. Data of first (red circles, n=1020) and second (blue dots, n=114) interventions together with a least square regression analysis with 95% confidence limits are plotted (Proc Gplot, SAS). In the insert, the box and whiskers plots of depths and volumes of first and second interventions illustrate the absence of differences.

**Figure 3:**
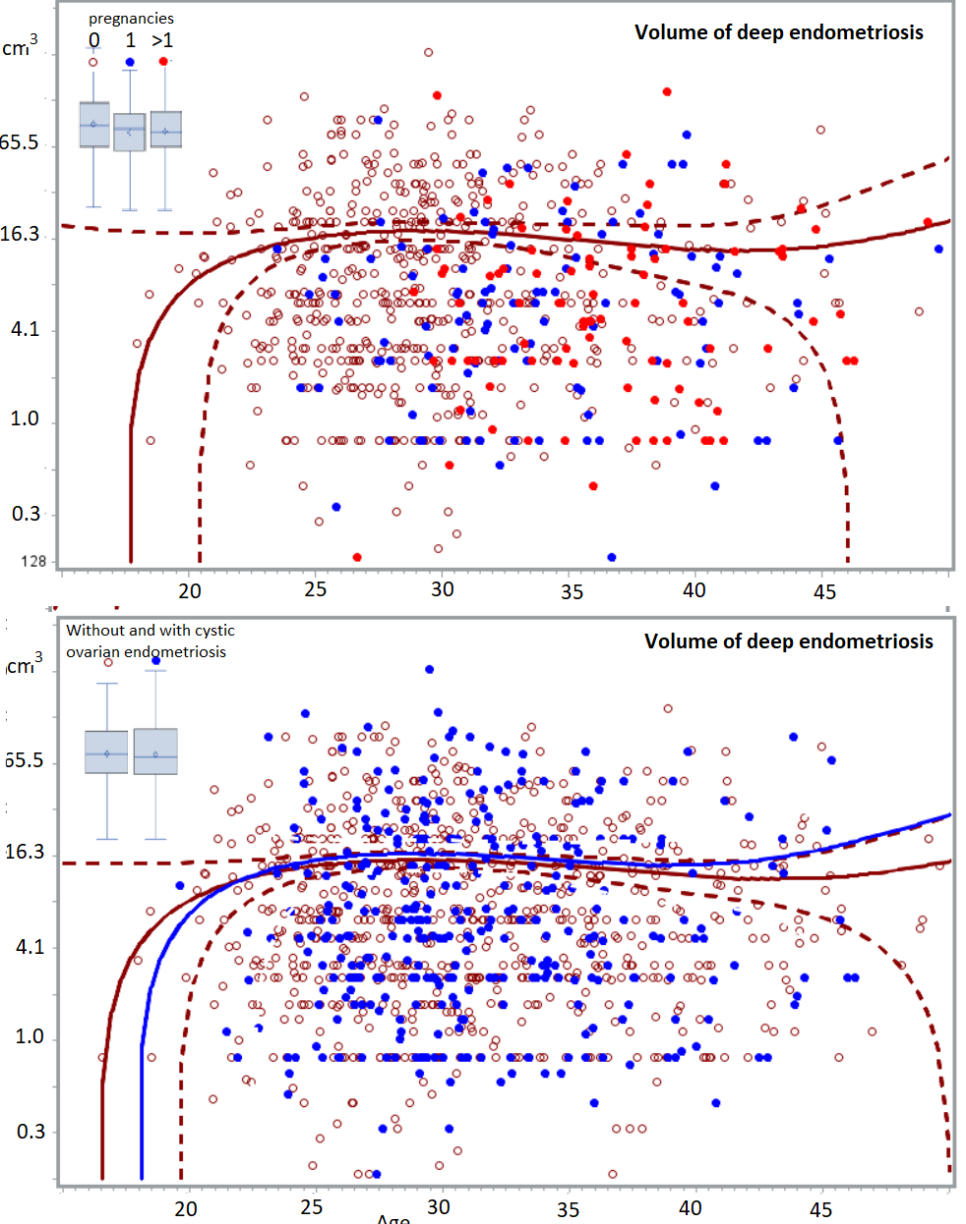
The volume of deep endometriosis lesions (cm^3^) does not vary between 24 and 50 years, for women without (red circle, n=547), with one (blue dots, n=114) or more than 1 child (red dots, n=93). The least-square regression analysis with 95% confidence limits indicate women without children. In the insert, the boxplots of women with 0, 1 or >1 children demonstrated the absence of a difference between the 3 groups. In the lower graph volumes of deep endometriosis are shown in women with (blue dots, n=344) and without (red circles, n=742) a cystic ovarian endometrioma. The boxplots highlight the absence of a significant difference.

**Figure 4:**
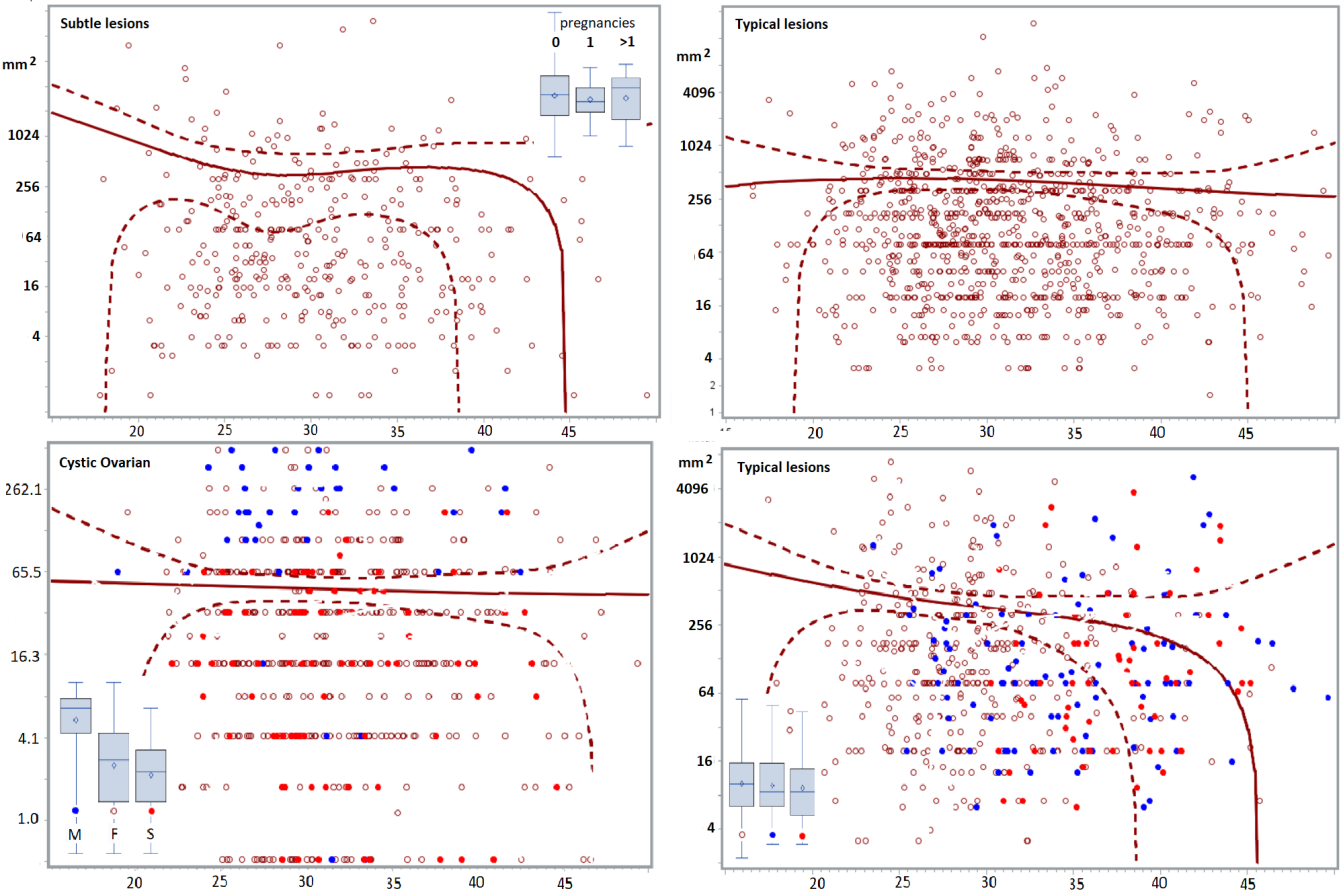
Pelvic area of subtle (n=363) and typical (n=2802) endometriosis lesions and volume of cystic ovarian endometriosis (n=601 ). No differences were found between women with 0, 1 and more than 1 previous pregnancies as evidenced from the boxplots for subtle and the Scatchard plots and boxplots of typical lesions. Scatchard plots of all typical lesions and of the typical lesions with 0.1 and >1 pregnancies are listed separately since pregnancy information was missing in almost half of the women. Not surprisingly, women with previous pregnancies were slightly older. Also between first and second surgeries, no differences were found for subtle, typical, and cystic ovarian endometriosis. The graph and boxplot of cystic ovarian endometriosis illustrates also the large volume of women undergoing a marsupialisation of cystic ovarian endometriosis.

Scatchard plots of individual lesions, do not suggest hidden subgroups. In adult women, the severity of lesions was highly variable for all types of endometriosis lesions, but severity did not increase with age. Deep lesions (n=1140) range from less than 0.5 cm 3 to more than 65 cm 3 , and the quadratic regression line of the whole group seems horizontal with narrow 95% confidence limits between 25 and 44 years of age. The volume and the depth of the deepest lesion were not different in women undergoing a first (n=1020) or a second intervention (n=114) (Figure 2) and in women with no (n=547), 1 (n=114) or more than 1 (n=93) previous pregnancies (Figure 3). The volume of deep endometriosis lesions also was not different between women with and without an associated cystic ovarian endometriosis (Figure 3). Also, the severity of subtle, typical (n=1008) and cystic ovarian (n=1008) endometriosis was highly variable and did not increase with age. Severity also was not less in women with a previous pregnancy or in women undergoing a second intervention (Figure 4).

In women younger than 22 years, the depths and volumes of deep endometriosis seemed lower. However, the wide confidence limits, the low numbers and high variability should make us cautious to conclude that this decline is significant. However, in the group less than 28 years old, the age of the women correlated (Spearman) with the depth of the deepest lesion (P<0.0001), with the total volume of deep endometriosis (P=0.0009) and the number of typical lesions (p=0.05), but not with the largest diameter or the total volume of cystic ovarian endometriosis.

**Figure 5:**
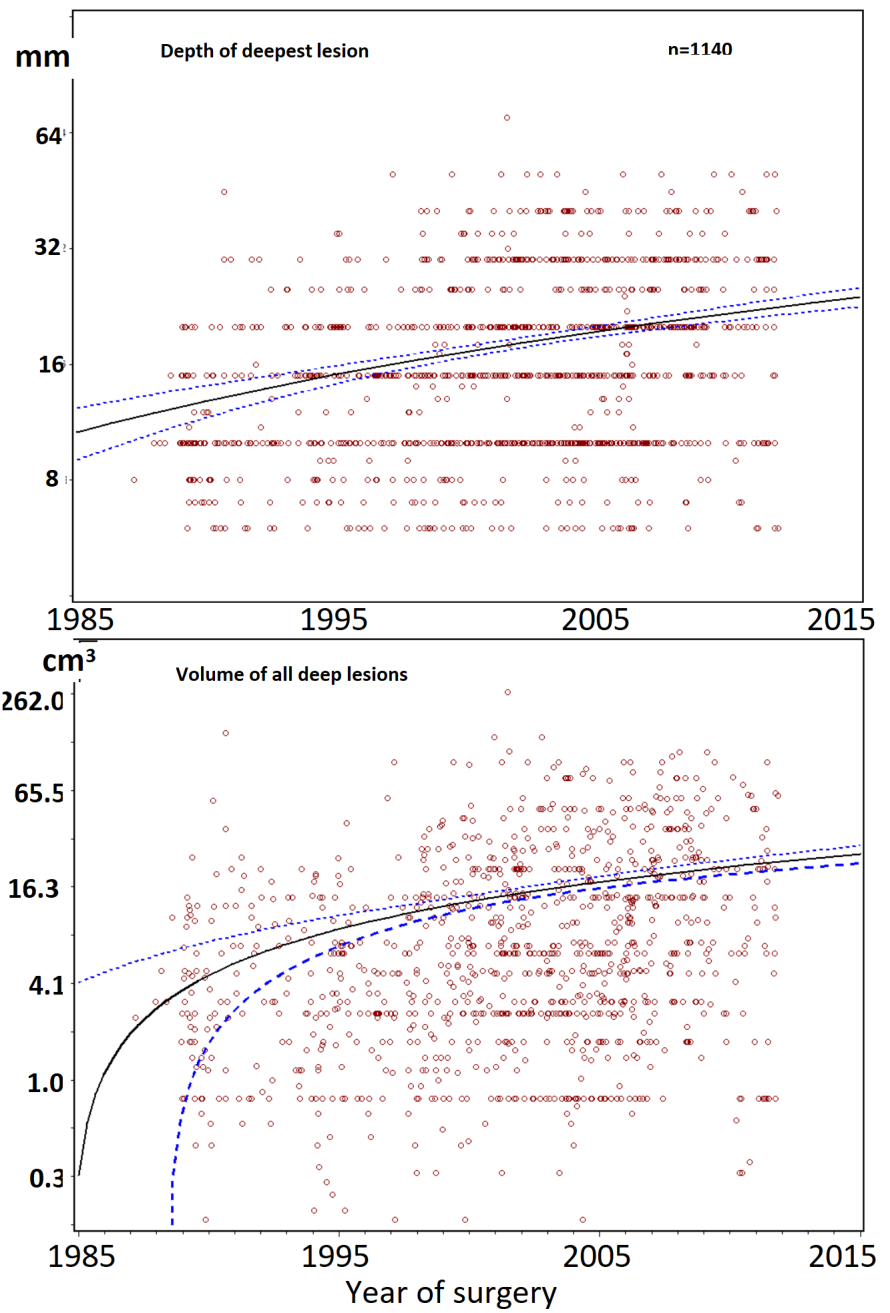
Depth of deepest lesion and volume of deep endometriosis by year of intervention.

To exclude that results were affected by a referral bias over the years, the distribution of volumes of cystic ovarian endometriosis, the depth and volumes of deep endometriosis (Figure 5) and the total area of subtle and of typical endometriosis were calculated for the years of surgery between 1988 and 2011. Depth and volume of deep endometriosis and diameter of cystic ovarian endometriosis increased slightly (P<0.001 Pearson). The areas of subtle endometriosis and typical endometriosis remained constant between 1988 and 2011. Also, the variability of typical, cystic and deep endometriosis did not vary over the years.

## Discussion

Interpretation of these data and clinical conclusions should be done carefully. Since in large datasets minor differences easily reach significance, the absence of significant differences is more likely to reflect reality. Second, the frequency distribution of lesions and the Scatchard plots of individual data demonstrate that between 24 and 44 years of age, the relative frequencies and severity of lesions remain constant. Before that, the frequency distribution and the Scatchard plots suggests an increase of severe lesions up to age 22 or age 24 or eventually up to age 26. Our numbers are too low to permit conclusions for ages beyond 44 years.

The severity and variability of each type of endometriosis are remarkably constant in adult women. This suggests that the growth of endometriosis lesions is limited after a variable period of growth. This concept that growth does not continue, is consistent with the clinical observation that most deep endometriosis lesions are no longer rapidly progressive when diagnosed clinically. However, the concept of limited growth of most endometriosis lesions does not exclude that the growth rate and duration of growth can be highly variable as suggested by the enormous variability in the severity of lesions. This is also consistent with the clinical observations that during surgery some deep endometriosis lesions look more proliferative and glandular while others are more fibrotic. The mechanism of this limited growth is unknown, but we can speculate that local factors such as immunology, immunologic maturation (Brenhouse and Schwarz, 2016) or a different set of GE incidents or driver mutations or repeated trauma and fibrosis with decreased vascularisation may play a role. Also, the clinical consequences of this limited growth are unclear. We do not know whether lesions are mainly symptomatic when active and growing or whether they remain symptomatic because of fibrosis even when no longer progressive.

It is surprising that the severity of lesions is not less in women with previous pregnancies, and that the severity of lesions is not different between the first and second intervention. Growth might only be affected during the pregnancy and continue thereafter. An alternative explanation is that the period of growth of endometriosis lesions is rather short and that pregnancies have little effect on existing lesions or on the initiation of new lesions after a pregnancy. A rather short period of growth could explain the absence of differences between a first and second surgery, and is consistent with the observation that many women operated for deep endometriosis, clearly recall when pain symptoms increased, which varied from a few months, up to 4 to 10 years. Unfortunately, exact data on initiation of pain before surgery and of previous medical treatment were not available in the database.

**Figure 6:**
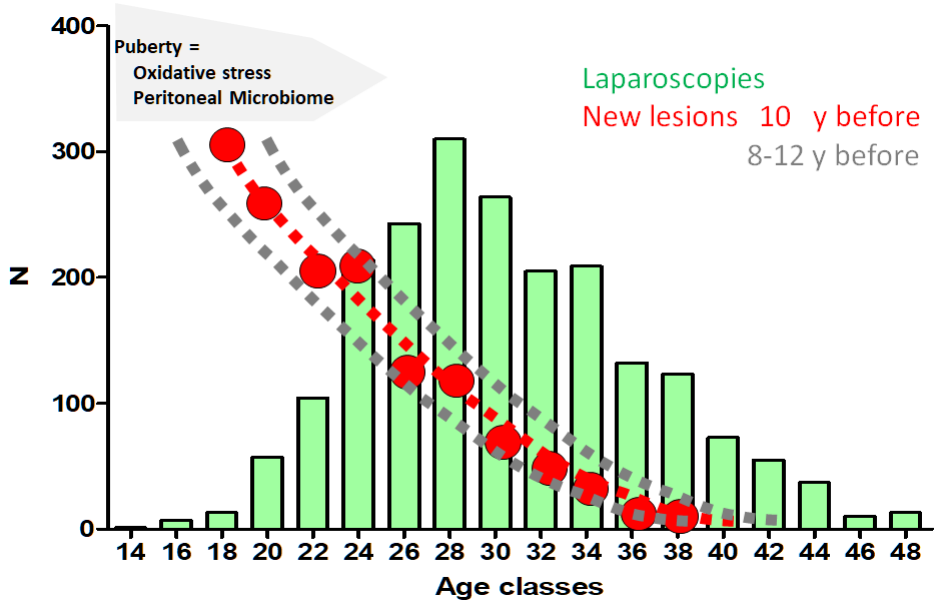
The risk of developing endometriosis decreases exponentially after puberty if the number of laparoscopies (green bars) would reflect the initiation of new lesions (red dots) 10 years earlier. A period of 10 years between the initiation of lesions and the laparoscopy was used since lesions need to grow for some period of time before becoming symptomatic and since the diagnostic delay up to 10 years. Women with a higher risk because of heredity, oxidative stress or infection may be more likely to develop the disease earlier in life, and the remaining group thus would have a lower risk.

The number of laparoscopies performed for endometriosis-associated pain increases from puberty up to age 28 and decreases thereafter. These data are comparable with the observation of laparoscopies for endometriosis in Germany (Haas et al., 2012) demonstrating an increasing rate of laparoscopies up to 30-35 years of age in women with confirmed endometriosis in Germany. The difference with our data could be due to the differences between referral centres, and global data in a country with probably an underestimation of subtle lesions. Considering that after initiation, endometriosis lesions need to grow to become symptomatic and considering the well known variable diagnostic delay before a laparoscopy is performed (Schenken, 2006; Hudelist et al., 2012; Chantalat et al., 2017; van der Zanden et al., 2018) the interval between the onset of endometriosis lesions and the laparoscopy, can be estimated to vary up to more than 10 years. Puberty and menarche suddenly change and probably increase a women’s risk of initiating endometriosis. As contributing factors, we may consider oestrogens and endocrinology, an immature immune system (Brenhouse and Schwarz, 2016), retrograde menstruation with implantation of cells and increased oxidative stress and sexual activity with the associated infection risk (Koninckx et al., 2019b). Similar to the observation that fertile women become pregnant more rapidly and that the remaining group thus becomes progressively less fertile, we suggest that in susceptible women the risk of initiating endometriosis is highest after menarche or after initiating sexual activity and that in the remaining group the risk decreases progressively. This is compatible with the decreasing risk of recurrences of cystic ovarian endometriosis with age (Liu et al., 2007). Figure 6 visualises the assumption of a 10- year delay between the initiation of endometriosis and the laparoscopy. The increasing number of laparoscopies until age 28 and the decreasing number thereafter could be compatible with an initially increased risk, decreasing progressively, together with a variable growth of endometriosis lesions, a variable diagnostic delay and other variables as the observation that laparoscopy is more likely to be performed with a shorter diagnostic delay in women of reproductive age with infertility.

These concepts that growth of endometriosis is limited, and that the risk of initiating is highest during adolescence and decreases progressively thereafter, could be important for endometriosis management. It suggests that prevention of initiation should focus on the younger age groups. Equally important is that delaying surgery might be less harmful when the growth of lesions is limited. It also emphasises how important it is to understand the growth potential of lesions and the effect of medical therapy on growth.

The strength of these observations is that the data were obtained in a single centre by a single surgeon without major changes in the indication and technique of surgery over the 20 years of observation. However, several biases exist. Girls below 14 years of age are absent from this series since in Leuven, patients up to age 14 are seen by paediatricians and referred to the department of surgery. A second bias is that the intake of medical therapy for a variable duration of time before surgery cannot be excluded (Schenken, 2006; Hudelist et al., 2012; Chantalat et al., 2017; van der Zanden et al., 2018). A third bias is a referral bias with relatively more severe endometriosis since Leuven University Hospital Gasthuisberg was a referral centre for deep endometriosis since the early nineties (Cornillie et al., 1990). We interpret the increasing volumes of deep endometriosis between 1988 and 2011 as a referral bias, although a real increase in the severity of endometriosis cannot be excluded.

In conclusion, the growth of endometriosis lesions seems to stop spontaneously as suggested by the constant severity of all types of endometriosis in adult women, at least between 24 and 44 years of age. Surprisingly, the severity is not less after previous pregnancies. Considering that the time needed for lesions to grow and become symptomatic plus the well known diagnostic delay, will be variable up to more than 10 years, the decreasing number of laparoscopies in women after 28 years, is compatible with a decreasing risk of initiating endometriosis after adolescence.
